# A novel BCR-ABL1 fusion gene identified by next-generation sequencing in chronic myeloid leukemia

**DOI:** 10.1186/s13039-016-0257-5

**Published:** 2016-06-27

**Authors:** Xiaodong Lyu, Jingke Yang, Xianwei Wang, Jieying Hu, Bing Liu, Yu Zhao, Zhen Guo, Bingshan Liu, Ruihua Fan, Yongping Song

**Affiliations:** School of Basic Medical Sciences, Zhengzhou University, Zhengzhou, Henan 450000 China; Central Laboratory, the Affiliated Cancer Hospital of Zhengzhou University; Henan Cancer Hospital, Zhengzhou, Henan 450000 China; Department of Hematology, the Affiliated Cancer Hospital of Zhengzhou University; Henan Cancer Hospital, Zhengzhou, Henan 450000 China

**Keywords:** BCR-ABL1, Next-generation sequencing, Chronic myeloid leukemia, SH3 domain, Tyrosine kinase, Imatinib

## Abstract

**Background:**

*BCR*-*ABL1* fusion proteins contain constitutively active tyrosine kinases that are potential candidates for targeted therapy with tyrosine kinase inhibitors such as imatinib in chronic myeloid leukemia (CML). However, uncharacterized *BCR*-*ABL1* fusion genes can be missed by quantitative RT-PCR (qRT-PCR)-based routine screening methods, causing adverse effect on drug selection and treatment outcome.

**Case presentation:**

In this study, we demonstrated that the next-generation sequencing (NGS) can be employed to overcome this obstacle. Through NGS, we identified a novel *BCR-ABL1* fusion gene with breakpoints in the *BCR* intron 14 and the *ABL1* intron 2, respectively, in a rare case of CML. Its mRNA with an e14a3 junction was then detected using customized RT-PCR followed by Sanger sequencing. Subsequently, the patient received targeted medicine imatinib initially at 400 mg/day, and later 300 mg/day due to intolerance reactions. With this personalized treatment, the patient’s condition was significantly improved. Interestingly, this novel fusion gene encodes a fusion protein containing a compromised SH3 domain, which is usually intact in the majority of CML cases, suggesting that dysfunctional SH3 domain may be associated with altered drug response and unique clinicopathological manifestations observed in this patient.

**Conclusion:**

We identified a novel *BCR-ABL1* fusion gene using NGS in a rare case of CML while routine laboratory procedures were challenged, demonstrating the power of NGS as a diagnostic tool for detecting novel genetic mutations. Moreover, our new finding regarding the novel fusion variant will provide useful insights to improve the spectrum of the genomic abnormalities recognizable by routine molecular screening.

## Background

Philadelphia translocation, the recurrent genomic rearrangement between chromosome 22q11 and 9q34, is a cytogenetic hallmark of Chronic myeloid leukemia (CML) [1]. It usually arises between genes *BCR* and *ABL1* in the intron regions, resulting in the formation of *BCR-ABL1* fusion genes and accordingly in the expression of chimeric gene transcripts with a juxtaposition of e13-a2, e14-a2, e19-a2, or e1-a2 [1]. These well characterized mRNA molecules, existed in three principal forms (p210, p190, and p230), have been screened routinely by multiplex quantitative RT-PCR (qRT-PCR) assays for diagnostic purpose in CML patients. However, these screening procedures have limitations as they were designed for detecting those previously characterized BCR-ABL1 fusion transcripts [2–14]. A false negative result may occur if a novel fusion mRNA does not contain sequences recognizable by primers within commercial or laboratory-developed screening kits, conferring a negative impact in treatment selection and therapeutic outcome.

To overcome these challenges, new technologies are in debt to be developed. The next-generation sequencing (NGS) is a powerful tool for detecting genomic abnormalities that are both known and previously uncharacterized [15, 16]. Due to improved efficiency and affordability, both whole genome sequencing (WGS) and whole exome sequencing (WES) in NGS platforms have become increasingly popular as alternative methods for detecting unknown and complex pathogenic mutations in cancer and other diseases [17, 18]. Additionally, targeted NGS applications have also been developed for known mutations, further facilitating NGS as a diagnostic tool for daily practice [18].

A CML case was presented in the current study. While both cytogenetic analysis and Fluorescent In-Situ Hybridization (FISH) suggested a Philadelphia translocation, Fluorescence one-step multiplex qRT-PCR, a routinely used screening method, failed to identify any *BCR-ABL1* transcripts. To resolve the discrepancy, we performed genomic sequencing using a WGS in a NGS platform and identified a novel *BCR-ABL1* fusion gene variant. The related transcript was then detected using RT-PCR with customized primers followed by Sanger sequencing. Our result should provide novel insights in BCR-ABL1 diagnosis and facilitate the utilization of NGS as an alternative or daily diagnostic tool in leukemia.

## Methods

### Detection of BCR-ABL1 gene rearrangement

Fluorescence one-step RT-PCR was performed as described previously [19]. In brief, total RNA was extracted from bone marrow aspiration samples with RNeasy Kit (Qiagen, CA, USA) according to vendor’s instruction and then treated with DNase I (DNA-*free*; Applied Biosystems-Ambion, TX, USA) to remove any DNA contamination. The one-step RT-PCR was then performed using Leukemia Related Fusion Gene Detection Kit for *BCR-ABL* p210, p190, or p230 (Yuanqi Bio-Pharmaceutical, Shanghai, China). For each reaction, 3 μl total RNA was mixed with 20 μl multiplex RT-PCR Buffer and 2 μl multiplex Enzyme mix in a total volume of 25 μl. The RT-PCR was conducted at 42 °C for 30 min, 94 °C for 5 min, and followed by 40 cycles 94 °C for 15 s and 60 °C for 1 min on a 7300 Real Time PCR System (ABI, USA).

### FISH analysis detecting BCR-ABL fusion

We conducted FISH analysis on patient’s bone marrow aspiration sample using *BCR-ABL* FISH Probe kit (GP Medical, Beijing, China) per vender’s instruction. Two DNA probes targeting the 5′ end of *BCR* and the 3′ end of *ABL1* were labeled with green and red fluorescent dye, respectively. In a normal nucleus, the probe-targeted sequences were located on different chromosomes as indicated as separated green and red signals. However, when fusion occurs in a tumor cell, a yellowish signal should be observed due to the fusion of *BCR* and *ABL1* genes, resulting in the colocalization of the green and red fluorescent signals.

### Whole genome sequencing in a next-generation sequencing platform

Genomic DNA (gDNA) library for next-generation sequencing was prepared using TruSeq Nano DNA Library Preparation Kit (Illumina, San Diego, CA). The gDNAs were shared into fragments using Covaris and ligated at both ends with indexed paired-end adaptors (Covaris, Woburn, MA, USA). The adapter-ligated gDNAS were purified and then used as templates in a ligation-mediated PCR for 8 cycles. The PCR amplicons were quantitated using Qubit 3.0 Fluorometer (Life Technologies) after removing primers, and then diluted into solutions at 0.75 ng/ul. The magnitude of enrichment were subsequently estimated using an Agilent 2100 Bioanalyzer (Agilent Technologies). Paired-end DNA sequencing was performed on a HiSeq X (Illumina, San Diego, CA). Illumina bcl2fastq software version 2.15 was used for base calling analysis.

## Case presentation

### Patient

**Fig. 1:**
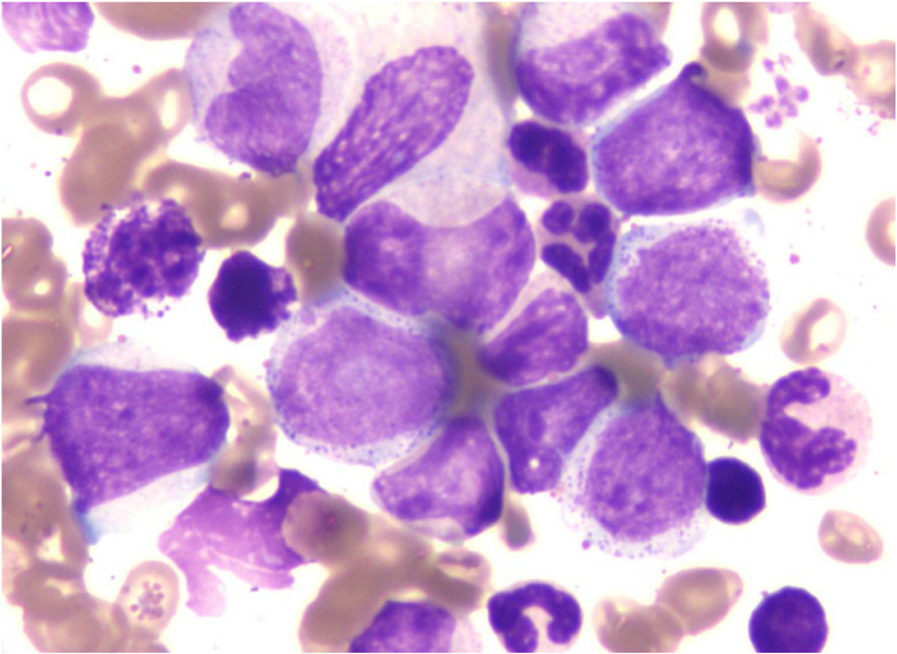
Image of bone marrow aspiration (400x) showing hypercellularity with elevated level of myeloblasts, eosinophils, and basophils

A 24-year-old male patient presented to the hospital with significant bilateral cervical lymphadenopathy in February 2015. Multiple large necrotic lymph nodes was increased in size from 1 × 1.5 cm to 5 × 3 cm within 10 days. Thoracic computed tomography (CT) detected additional bilateral axillary lymphadenopathy. The patient also showed symptoms including constant low fever, red and swollen gums, and splenomegaly. The patient was diagnosed as CML upon the completion of blood and bone marrow examininations. The full blood count indicated leukocytosis with elevated level of white blood cells (WBCs, 222 × 10^9^/L), neutrophils (95.3 × 10^9^/L), platelet (938 × 10^9^/L), and normal level of hemoglobin (116 g/L). Periphery blood smear analysis suggested granulocytosis with the presence of circulating myeloblasts (6 %) and immature granulocytes at various stages (Table 1). Bone marrow aspiration analysis indicated hypercellularity with prominent eosinophilia, basophilia, and elevated level of myeloblasts (5.4 %) and promyelocytes (8 %) (Table 1 and Fig. 1). A decreased level of lymphocytes with normal morphology was also observed. The myeloid/erythroid (M:E) ratio was significantly increased in both periphery blood and bone marrow (Table 1).

**Table 1:** Analysis of periphery blood smear and bone marrow aspiration

Cell type	Blood smear	Bone marrow aspiration	
	Cell count (%)	Reference (Mean ± sem)	Cell count (%)
Myeloblast	6.0	0.64 ± 0.33	5.4
Promyelocyte	4.0	1.57 ± 0.60	8.0
Neutrophil			
N. myelocyte	11.0	6.49 ± 2.04	18.2
N. metamyelocyte	8.5	7.90 ± 1.97	7.6
N. band	14.5	23.72 ± 3.50	14.6
Neutrophil	14.0	9.44 ± 2.92	13.8
Eosinophil	13.0	0.86 ± 0.61	11.0
Basophil	5.5	0.30 ± 0.05	1.8
Lymphocyte	16.0	22.78 ± 7.04	9.4
Monocyte	7.5	3.00 ± 0.88	4.2
M:E ratio	153:0	3.00 ± 1.00	215:1

### Detection of BACR-ABL1 fusion gene with the NGS

**Fig. 2:**
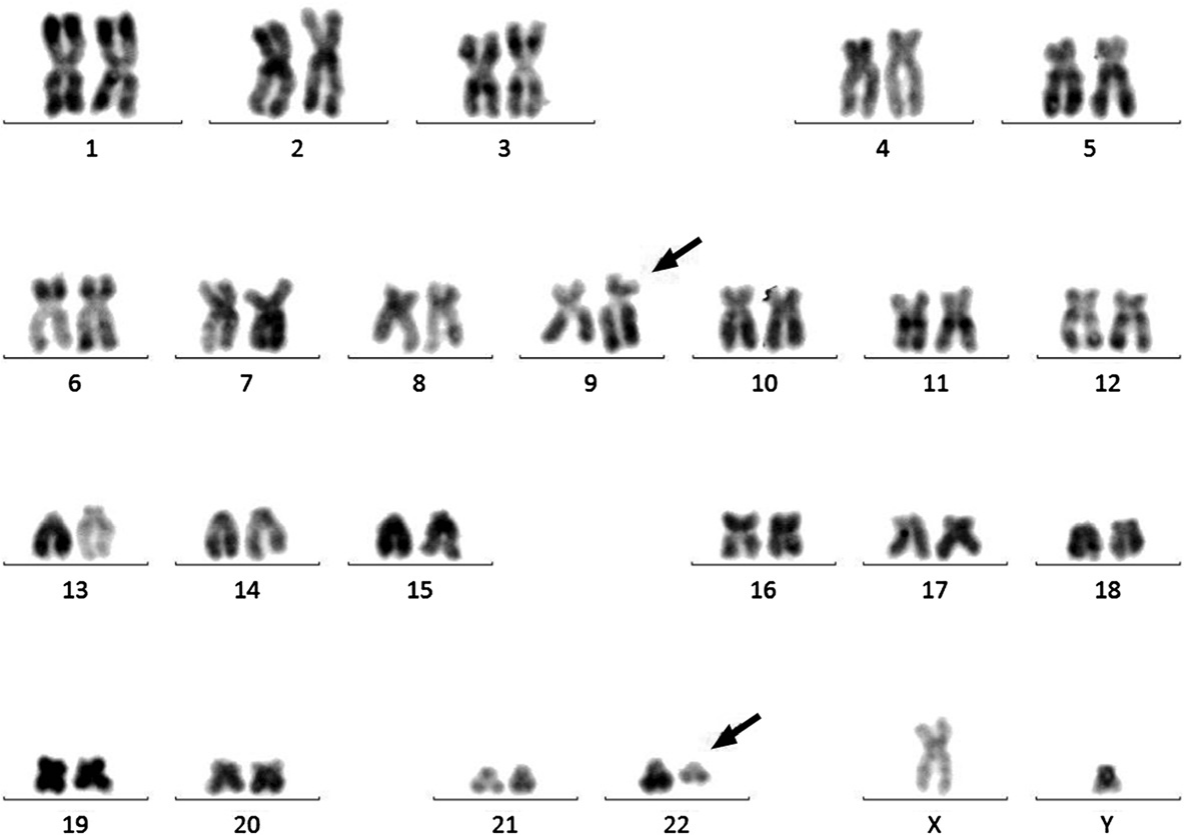
Cytogenetic analysis of bone marrow aspiration showing a karyotype 46,xy,t (9;22) (q34;q11). A gene translocation occurred between chromosomes 9 and 22 resulting Philadelphia chromosomes at the sites as indicated with *arrows*

**Fig. 3:**
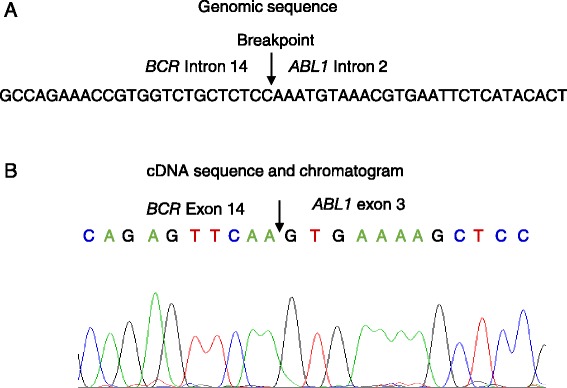
A novel BCR-ABL1 fusion gene and its transcript in a CML case. **a** Diagram showing the breaking point (or fusion junction) and flanking sequences from *BCR* Intron 14 and *ABL1* intron 2, which were identified using WGS in a NGS platform. **b** BCR-ABL1 cDNA sequence around the fusion junction and related chromatogram are shown. The junction is indicated with an *arrow*. The origins of cDNA sequences were also indicated

Bone marrow karyotype showed a phenotype of 46,XY,t (9;22) (q34;q11) [10/10] (Fig. 2). FISH analysis suggested a possible fusion between the *BCR* and *ABL1* genes. However, fluorescent quantitative RT-PCR (qRT-PCR) failed to detect any previously characterized *BCR*-*ABL1* fusion transcripts p210 (e13-a2 and e14-a2), p190 (e1-a2), and p230 (e19-a2). To clarify whether a *BCR*-*ABL1* fusion occurred in this patient, we conducted whole genome sequencing (WGS) analysis on a next-generation sequencing (NGS) platform. We detected a *BCR*-*ABL1* fusion gene with novel breakpoints in the *BCR* intron 14 and the *ABL1* intron 2, respectively (Fig. 3a). Based on this finding, we predicted that the fusion junction in the novel *BCR*-*ABL1* fusion transcript was flanked by sequences from the *BCR* exon 14 (e14) and *ABL1* exon 3 (a3), which were used as templates for designing customized PCR primers. Through using these primers, we conducted RT-PCR followed by Sanger sequencing and identified the corresponding *BCR*-*ABL1* hybrid mRNA with an e14a3 junction (Fig. 3b). Through NGS, we also detected nonsynonymous single nucleotide variant (SNV) in several well-known leukemic genes, including FLT3 (c.A20G:p.D7G), KIT (c.A1621C:p.M541L), PAX5 (c.C878T:p.T293), and TP53 (c.C215G:p.P72R). However, it is not clear whether these mutations play any important roles in leukemogenesis due to either no or limited reports associated with each of these mutations in cancer.

### Treatment

Diagnosed with CML, the patient was initially treated with hydroxyurea to improve blood count. Additional medicines, including antibiotics and anti-infection drugs, were also prescribed to relief fever, gum swelling and redness, and other complications. After detecting the novel *BCR*-*ABL1* fusion gene, imatinib was used at 400 mg/day as a targeted treatment. However, despite an improved blood count, the patient showed intolerant reactions, including raised fever, sore throat, right-side maxillofacial swelling, etc., after 40 days’ treatment. The dosage of imatinib was then reduced to 300 mg/day. The full blood count was significantly improved with a WBC count at a relatively stable level (3 to 10 × 10^9^/L). The patient was then released with continued imatinib usage at 300 mg/day and with periodic follow-up examinations.

## Discussion

In this study, we reported that a novel *BCR-ABL1* fusion gene was identified by NGS technology, but not by a routinely-used, RT-qPCR-based method in a rare case of CML. Its mRNA transcript was subsequently detected using RT-qPCR with customized primers. Our result suggested that the NGS-based application can be employed as a diagnostic tool in those difficult cases with novel or complex genomic abnormalities.

CML, a malignant myeloproliferative disorder, originates from a hematopoietic stem cell that carries a Philadelphia chromosome, characterized by a reciprocal translocation between chromosomes 9 and 22, or t (9,22). This translocation generates a fusion gene with the juxtaposition of the *BCR* gene fragment at the 5′ end and the *ABL1* gene fragment at the 3′end [20]. An in-frame *BCR-ABL1* fusion gene is likely oncogenic, which can be transcribed into *BCR*-*ABL1* fusion mRNA and translated into BCR-ABL1 fusion protein. As discovered by genomic and cDNA sequencing in CML patients, the breakpoint in the *ABL1* gene is usually detected in a region between exon 1a and exon 2 [21]. In rare occasions, the breakpoints were detected in intron region downstream to the exon 2, resulting in a smaller fusion gene skipping exon 2 [22]. In the *BCR* gene, the breakpoints are most frequently detected in introns between exons 13 and 14 or exons 14 and 15 within a region recognized as the major breakpoint cluster region (M-*bcr*) covering exons 12–16 (e12-e16, or historically known as b1-b5) [1]. The encoding proteins were termed based on their molecular weight as p210^BCR-ABL1^ fusion proteins with junctions described as e13a2 or e14a2. In rare cases, the breakpoints were found in the minor bcr (m-bcr) between the two alternative exons e2′ and e2 [23] or μ-bcr between exons 19 and 20 [24–26]. Their encoding proteins were described as p190^BCR-ABL1^ and p230^BCR-ABL1^.

In this study, we reported a CML patient with constant low fever, swollen gums, bilaterally cervical lymphadenopathy, and splenomegaly. Interestingly, this patient showed otherwise normal physical and mental conditions. Upon blood and bone marrow tests, this patient was diagnosed as CML. Further cytogenetic analysis confirmed the existence of Philadelphia chromosome. However, routine fluorescent RT-qPCR screening cannot detect any form of *BCR-ABL1* fusion mRNAs that were previously characterized and detected in the majority of CML cases. To resolve the discrepancy, we performed WGS on an Illumina HiSeq X and identified a novel *BCR*-*ABL1* fusion gene. Although the breakpoint in the *BCR* gene is located in intron between exons 13 and 14 within the M-bcr region, the breakpoint in the *ABL1* is novel, located in intron 2, a region downstream to the exon 2. The resulting fusion gene is presumably transcribed into a smaller fusion mRNA with an e14a3 junction, skipping the entire exon 2 (174 nucleotides). This novel transcript was initially undetectable by routine RT-qPCR-based diagnostic screening as this type of fusion transcripts have only been reported in rare cases and have not been the subjects for detection using RT-PCR-based routine *BCR-ABL1* screening [22]. This rare fusion variant was only reported in 4 leukemia cases, including one case of CML [27] and three cases of acute lymphoblastic leukemia (ALL) [28–30], Unlike our study, none of these studies performed genomic sequencing to show the existence of this fusion gene on the genomic level. After NGS application, this novel fusion mRNA (cDNA) was eventually identified using RT-PCR with a pair of customized primers targeting the *BCR* exon 14 (e14) and *ABL1* exon 3 (a3), respectively, and followed by Sanger sequencing of PCR amplicons. Similar to our study, a recent publication has shown that an e14a3 transcript was detected by a customized PCR using primers targeting the *BCR* exon e13 and the *ABL1* exon a3, but not by the PCR using conventional primer sets [28]. Thus, if a *BCR-ABL1* negative result was obtained by conventional screening, care should be taken to avoid misdiagnosis, which may cause an adverse impact on therapeutic outcome. The correct diagnosis will allow physicians to make right decisions whether a targeted therapy (imatinib in this case) or other treatment plans should be used for better outcome achievement.

ABL1 protein is a nonreceptor tyrosine kinase containing a tyrosine kinase domain. The fusion with the *BCR* gene creates a fusion protein with an constitutively active ABL1 tyrosine kinases, which are believe to be causative in CML [31]. The majority of BCR-ABL1 proteins contain juxtaposed SH3-SH2 domains, which are located upstream to the tyrosine kinase domain and regulate kinase activity through their interactions with other proteins [32]. It has been shown that the interaction between BCR-ABL1 SH3 and RAD51 proline-rich regions caused RAD51 phosphorylation, resulting in secondary chromosomal aberrations that contribute to disease progression and relapse in CML [33]. In contrast, small protein apoptin, a tumor-selective killer [34], is a negative regulator of BCR-ABL1 kinase via its ability to interact with BCR-ABL1 SH3 domain [35]. Moreover, it has also been suggested that sensitivity to the ABL TKIs is altered by either SH3-SH2 mutations or the interaction between ABL1 SH3-SH2 and other proteins such as RIN1 [36, 37]. SH3 domain is encoded by ABL1 exons 2 and 3. In our case, the whole exon 2 sequence including 51 nucleotides used for encoding the part of the ABL1 SH3 domain, were excluded in this novel *BCR*-*ABL1* fusion gene. Thus, this fusion gene is predicted to encode a fusion protein with a compromised SH3 domain. It is not clear whether this SH3 domain truncation is associated with some of the less common clinical presentations and drug response including intolerance to the normal dose of imatinib. Further studies are warranted to clarify these questions.

Through NGS, we also detected nonsynonymous mutations in leukemic genes *TP53*, *FLT3*, *KIT*, and *PAX5*, which were co-existed with BCR-ABL1 fusion. This result suggested that this CML is highly heterogeneous. *TP53,* the most frequently mutated gene in cancer, has often been detected with mutation in acute myeloid leukemia (AML), ALL, and chronic lymphocytic leukemia (CLL), predicting poor prognosis [38–40]. Both *FLT3* and *KIT* mutations have been shown to be associated with poor prognosis in AML [41, 42], while mutation in the gene *PAX5* are considered as signature mutations in precursor B-ALL [43, 44]. Although all these genes are frequently mutated in leukemia, the particular *FLT3* and *PAX5* mutations identified in this CML patient have never been reported before in any type of cancer, while the *TP53* and *KIT* mutations were only described in prostate cancer and melanoma [45, 46]. It should be noted that these novel mutations would be less likely to be detected by routine PCR-based molecular screening due to their novelty. Meanwhile, it remains to be determine whether these mutations are recurrent and play any pathological role in CML.

## Conclusions

Through NGS application, we have identified a novel *BCR-ABL1* fusion (with an e14a3 junction) that is undetectable by routine qRT-PCR-based laboratory procedures in a rare case of CML. Due to the correct diagnosis, the physicians prescribed targeted therapy using imatinib to this patient, leading to a better treatment outcome. Our NGS result also suggested that this fusion gene encodes a protein containing a truncated SH3 domain, which may contribute to the unique clinical presentation and altered drug response in this patient. Moreover, novel nonsynonymous mutations in the *TP53*, *FLT3*, *KIT*, and *PAX5* genes, are co-existed with BCR-ABL1 fusion, indicating the high heterogeneity in this disease. Therefore, this study has demonstrated the value of NGS as a powerful diagnostic tool for identifying novel and complex genetic mutations.

## Abbreviations

CML, Chronic myeloid leukemia; FISH, Fluorescent In-Situ Hybridization; NGS, Next-generation sequencing; TKI, Tyrosine kinase inhibitor; WES, Whole exome sequencing; WGS, Whole genome sequencing
